# *Sargassum fusiforme* Polysaccharides Prevent High-Fat Diet-Induced Early Fasting Hypoglycemia and Regulate the Gut Microbiota Composition

**DOI:** 10.3390/md18090444

**Published:** 2020-08-27

**Authors:** Bin Wei, Qi-Wu Zhong, Song-Ze Ke, Tao-Shun Zhou, Qiao-Li Xu, Si-Jia Wang, Jian-Wei Chen, Hua-Wei Zhang, Wei-Hua Jin, Hong Wang

**Affiliations:** 1 College of Pharmaceutical Science & Collaborative Innovation Center of Yangtze River Delta Region Green Pharmaceuticals, Zhejiang University of Technology, Hangzhou 310014, China; binwei@zjut.edu.cn (B.W.); zqw17816038275@163.com (Q.-W.Z.); kesongzeke@163.com (S.-Z.K.); zhoutaoshun@yeah.net (T.-S.Z.); liujiahui0829@163.com (Q.-L.X.); new8090@hotmail.com (S.-J.W.); cjw983617@zjut.edu.cn (J.-W.C.); hwzhang@zjut.edu.cn (H.-W.Z.); 2 Center for Human Nutrition, David Geffen School of Medicine, University of California, Rehabilitation Building 32-21, 1000 Veteran Avenue, Los Angeles, CA 90024, USA; 3 College of Biotechnology and Bioengineering, Zhejiang University of Technology, Hangzhou 310014, China

**Keywords:** *Sargassum fusiforme* polysaccharides, high-fat diet, gut microbiota, early fasting hypoglycemia

## Abstract

A low fasting blood glucose level is a common symptom in diabetes patients and can be induced by high-fat diet (HFD) feeding at an early stage, which may play important roles in the development of diabetes, but has received little attention. In this study, five polysaccharides were prepared from *Sargassum* *fusiforme* and their effects on HFD-induced fasting hypoglycemia and gut microbiota dysbiosis were investigated. The results indicated that C57BL/6J male mice fed an HFD for 4 weeks developed severe hypoglycemia and four *Sargassum* *fusiforme* polysaccharides (SFPs), consisting of Sf-2, Sf-3, Sf-3-1, and Sf-A, significantly prevented early fasting hypoglycemia without inducing hyperglycemia. Sf-1 and Sf-A could also significantly prevent HFD-induced weight gain. Sf-2, Sf-3, Sf-3-1, and Sf-A mainly attenuated the HFD-induced decrease in Bacteroidetes, and all five SFPs had a considerable influence on the relative abundance of *Oscillospira*, *Mucispirillum*, and *Clostridiales*. Correlation analysis revealed that the fasting blood glucose level was associated with the relative abundance of *Mucispinllum* and *Oscillospira*. Receiver operating characteristic analysis indicated that *Mucispinllum* and *Oscillospira* exhibited good discriminatory power (AUC = 0.745–0.833) in the prediction of fasting hypoglycemia. Our findings highlight the novel application of SFPs (especially Sf-A) in glucose homeostasis and the potential roles of *Mucispinllum* and *Oscillospira* in the biological activity of SFPs.

## 1. Introduction

Hyperglycemia is a dangerous and prevalent symptom in patients with diabetes, which has attracted extensive attention [1,2,3,4]. Hyperglycemia in animal models usually develops after long-term high-fat diet (HFD)-feeding and its progression may differ in different animal models [5,6]. A low fasting blood glucose (FBG) level, also called fasting hypoglycemia, is a common symptom in patients with diabetes and can be established in animal models after receiving short-term HFD-feeding. Fasting hypoglycemia is also a dangerous condition and may cause severe hypoglycemia symptoms, such as an irregular heartbeat and unconsciousness. A recent study suggests that FBG lower than 4.0 mmol/L is associated with an increased risk of many kinds of mortality, such as cardiovascular events and stroke [7]. Moreover, fasting hypoglycemia has also been reported in the early stage of HFD-fed mice. For example, He et al. reported that the FBG showed a decreasing tendency after 8 weeks of HFD feeding in C57BL/6J mice, and *Astragalus* polysaccharides could not reverse the decreasing tendency [8]. Unfortunately, HFD-induced early fasting hypoglycemia has not aroused too much attention.

*Sargassum fusiforme* is an edible brown algae and widely distributed in eastern Asian countries, including China, Japan, and South Korea. *S. fusiforme* has been developed as a traditional ethnic medicine for the treatment of thyroid disease and atherosclerosis [9], and polysaccharides, phlorotannins, and meroterpenoids have been identified as the major components underlying its pharmacological properties [10]. *S. fusiforme* polysaccharides (SFPs) mainly consist of sulfate fucoidan and exhibit multiple biological activities, including antioxidant, anti-tumor, immunity promoting, anti-aging, anti-hypoglycemia, anti-coagulation, and anti-bacteria activities [11,12,13,14]. For example, Cheng and co-workers found that the 6-week administration of *S. fusiforme* fucoidan exhibited a significant hypoglycemic effect and alleviated the pathological change in the heart and liver in HFD/streptozotocin (STZ)-induced diabetic mice, and the authors declared that the regulatory effect of *S. fusiforme* fucoidan on the fecal microbiota is a potential mechanism for attenuating the symptoms of diabetes [12]. However, the effects and underlying mechanisms of SFPs in HFD-induced early fasting hypoglycemia and gut microbiota dysbiosis, which are critical for their potential application, have not been investigated.

Therefore, in the present study, five SFPs were prepared from *S*. *fusiforme* using different extraction methods and the physicochemical properties of the SFPs were characterized using our previously established approaches [15]. Then, the effects and underlying mechanisms of the SFPs in HFD-induced early fasting hypoglycemia and gut microbiota dysbiosis were explored.

## 2. Results

### 2.1. Preparation and Physicochemical Properties of SFPs

Through hot-water and acid extraction methods, five polysaccharide fractions, including Sf-1, Sf-2, Sf-3, Sf-3-1, and Sf-A, were obtained from *S. fusiforme*. The total sugar, sulfate group, protein, and uronic acid contents, as well as the average molecular weight and monosaccharide composition, of these five SFPs are summarized in Table 1. The results showed that all of them have high total sugar (51.9–80.3%) and low protein (0.5–2.4%) and uronic acid (0.1–7.7%) contents. Sf-3 derived from the 2.0M NaCl eluent has the highest sulfate content (35.1%), which was consistent with the separation mechanism of diethylaminoethyl (DEAE) anion-exchanging chromatography. The average molecular weight of the five polysaccharides (Sf-1, Sf-2, Sf-3, Sf-3-3, and Sf-A) was detected by High-Performance Size Exclusion Chromatography (HPSEC) and was determined to be 698.3/8.9, 95.5/9.5, 229.5, 10.0, and 46.5/5.1 kDa, respectively (Table 1 and Figure S1), suggesting the wide range of SFPs in terms of the molecular weight.

As shown in Table 1, these five SFPs were mainly composed of mannose, galactose, glucose, glucuronic acid, and fucose. Specifically, Sf-1 mainly consisted of glucose, suggesting that Sf-1 was identified as laminaran. Sf-2 and Sf-A exhibited the same monosaccharide composition, but different molar ratios. The predominant monosaccharide in Sf-2 was fucose, while those in Sf-A were glucose and fucose (1.26:1). Sf-3 contained galactose and glucose at a ratio of 0.24:1. After Sf-3 was hydrolyzed by H_2_O_2_, the molecular weight of the hydrolysis product Sf-3-1 was significantly decreased and the monosaccharide composition was slightly changed. For example, the average molecular weight of Sf-3 was 229.5 kDa, while that of Sf-3-1 was only 10.0 kDa.

### 2.2. SFPs Significantly Alleviated HFD-Induced Early Fasting Hypoglycemia

**Figure 1:**
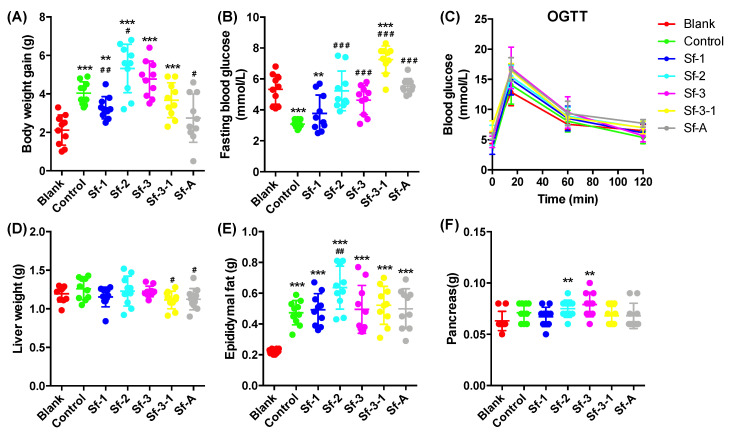
Effects of *S. fusiforme* polysaccharides on the (**A**) body weight, (**B**) fasting blood glucose, (**C**) oral glucose tolerance test (OGTT), (**D**) liver, (**E**) epididymal fat, and (**F**) pancreas weight in high-fat diet (HFD)-treated mice. Values are the mean ± SD (n = 10). * *p* < 0.05, ** *p* < 0.01, and *** *p* < 0.001 vs. blank. ^#^ *p* < 0.05, ^##^ *p* < 0.01, and ^###^ *p* < 0.001 vs. control.

To investigate the effects of SFPs on HFD-treated mice, C57BL/6J male mice were fed an HFD with or without SFP supplementation for 4 weeks. As shown in Figure 1A, a significant increase (90.6%, *p* = 0.00001) in body weight gain was found between the blank and control groups, indicating that 4-week HFD feeding led to a change in body weight. The administration of Sf-1 or Sf-A alleviated the increase in body weight by 35.8% (*p* = 0.009) and 61.3% (*p* = 0.011), respectively, while the administration of Sf-2 and Sf-3 further exacerbated the body weight gain (*p* = 0.012 and 0.054, respectively) (Figure S2). Interestingly, the FBG level in the control group decreased dramatically compared to the blank group after 4-week HFD feeding (42.2%, *p* = 0.00003), but unlike Sf-1, treatment with Sf-2, Sf-3, Sf-3-1, or Sf-A significantly reversed the decrease (*p* < 0.0004), without inducing hypoglycemia (Figure 1B). 

In an oral glucose tolerance test (OGTT), the blood glucose reached the maximal level at 15 min after the oral glucose load, and no significant difference was found between the blank and control groups. SFP administration had no beneficial effect on glucose tolerance (Figure 1C). HFD and polysaccharide administration showed a slight influence on liver and pancreas weights, while HFD dramatically increased the epididymal fat weight and SFP administration could not reverse the increase (Figure 1D–F).

### 2.3. SFPs Regulated the Gut Microbiota Composition

**Figure 2:**
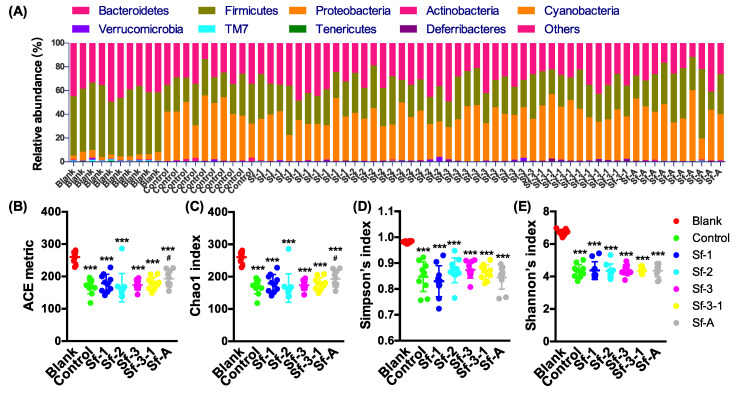
Effects of *S. fusiforme* polysaccharides on the composition and α-diversity of the gut microbiota in HFD-treated mice. (**A**) Gut microbiota composition at the phylum level, (**B**) Abundance-based Coverage Estimator (ACE) metric, (**C**) Chao1 diversity index, (**D**) Simpson’s diversity index, and (**E**) Shannon’s diversity index of the gut microbiota. Values are the mean ± SD (n = 10). *** *p* < 0.001 vs. blank. ^#^ *p* < 0.05 vs. control.

The effects of SFPs on the gut microbiota composition in HFD-treated mice were investigated by the 16S rRNA sequencing of cecum contents. As can be seen in Figure 2, the 4-week HFD dramatically altered the gut microbiota composition in C57BL/6J mice, decreasing the relative abundance of Firmicutes and α-diversity, and increasing the relative abundance of Proteobacteria. For example, all four α-diversity indices (Abundance-based Coverage Estimator (ACE) metric, and Chao1, Simpson’s, and Shannon’s diversity index) of the gut microbiota in the control group significantly decreased compared with the blank group (10.9–36.5%, *p* < 0.00005), but SFP administration displayed less influence on the α-diversity of the gut microbiota than HFD (Figure 2B–E).

**Figure 3:**
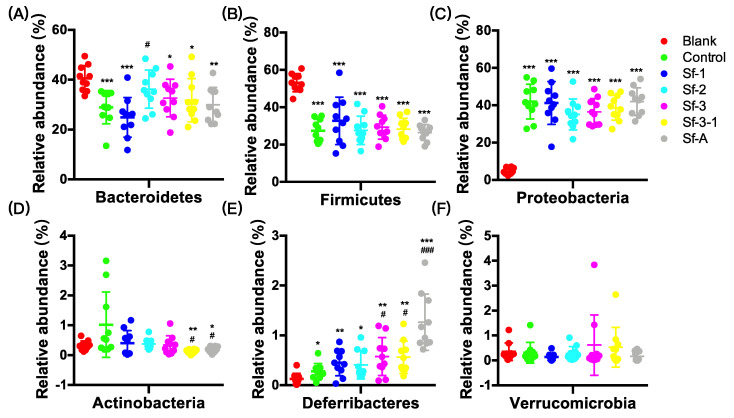
Effects of *S. fusiforme* polysaccharides on the relative abundance of (**A**) Bacteroidetes, (**B**) Firmicutes, (**C**) Proteobacteria, (**D**) Actinobacteria, (**E**) Deferribacteres, and (**F**) Verrucomicrobia in the gut microbiota in HFD-treated mice. Values are the mean ± SD (n = 10). * *p* < 0.05, ** *p* < 0.01, and *** *p* < 0.001 vs. blank. ^#^ *p* < 0.05, ^###^ *p* < 0.001 vs. control.

**Figure 4:**
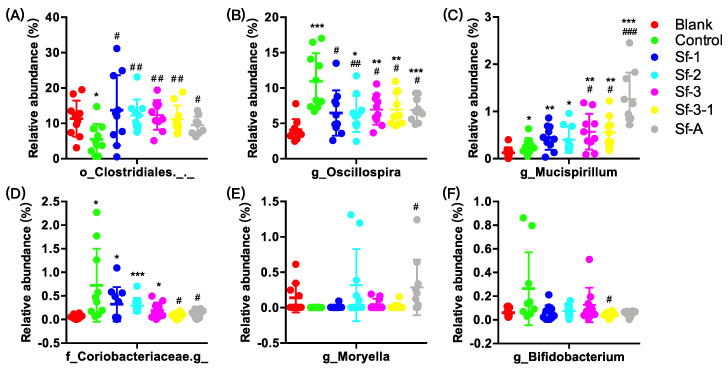
Effects of *S. fusiforme* polysaccharides on the relative abundance of (**A**) o_*Clostridiales*._._, (**B**) g_*Oscillospira*, (**C**) g_*Mucispirillum*, (**D**) f_*Coriobacteriaceae*.g_, (**E**) g_*Moryella*, and (**F**) g_*Bifidobacterium* in the gut microbiota in HFD-treated mice. Values are the mean ± SD (n = 10). * *p* < 0.05, ** *p* < 0.01, and *** *p* < 0.001 vs. blank. ^#^ *p* < 0.05, ^##^ *p* < 0.01, and ^###^ *p* < 0.001 vs. control.

The oral administration of SFPs mainly regulated the relative abundance of Actinobacteria (Figure 3). For example, at the phylum level, Sf-2, Sf-3, Sf-3-1, and Sf-A could reverse the increase in Actinobacteria (Figure 3D) compared with the control group. Notably, oral administration with Sf-2 also alleviated the decrease in Bacteroidetes (18.0%, *p* = 0.035) and increase in Proteobacteria (155.1%, *p* = 0.098) compared with the control group (Figure 3A,C). At the genus level, SFP administration mainly regulated the relative abundance of o_*Clostridiales*._._ (unidentified genus belonging to o_*Clostridiales*), g_*Oscillospira*, g_*Mucispirillum*, f_*Coriobacteriaceae*.g_(unidentified genus belonging to f_*Coriobacteriaceae*), g_*Moryella*, and g_*Bifidobacterium* (Figure 4). For example, all SFPs could alleviate the decrease in o_*Clostridiales*._._ (50.5–72.3%, *p* = 0.006–0.033), and the increase in g_*Oscillospira* (106.7–121.4%, *p* = 0.007–0.015) (Figure 4A,B). Moreover, SFPs also displayed selective enrichment in some less dominant species, including g_*Mucispirillum* and g_*Moryella* (Figure 4C,E).

### 2.4. SFPs Regulated the Metabolic Pathway Coverage of the Gut Microbiome

**Figure 5:**
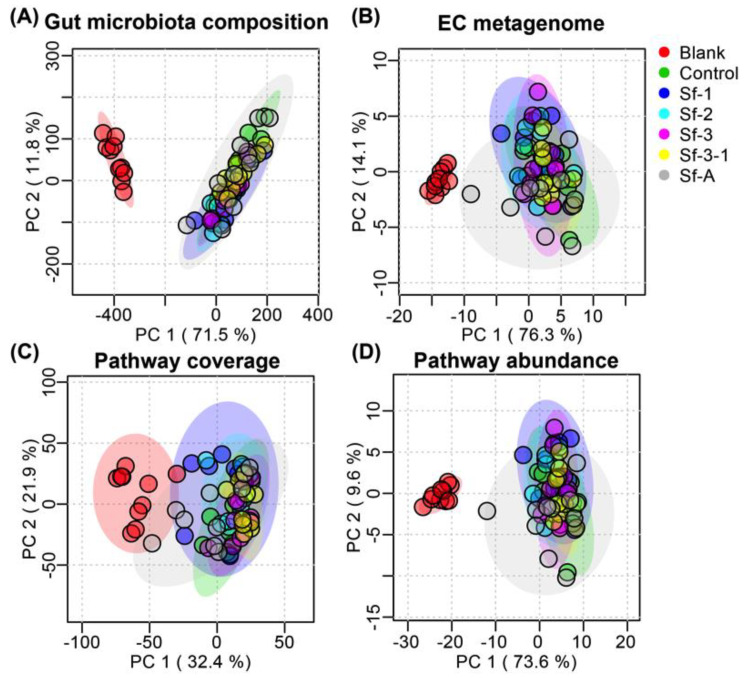
Effects of *S. fusiforme* polysaccharides on the (**A**) gut microbiota composition at the genus level, (**B**) predicted functional gene, (**C**) metabolic pathway coverage, and (**D**) abundance in HFD-treated mice.

To further understand the influence of SFPs on the structure and function of the gut microbiota, the functional gene, metabolic pathway coverage, and abundance were predicted using PICRUSt2 based on the 16S rRNA sequences. As shown in Figure 5A, the unsupervised principal components analysis (PCA(plot confirmed the fact that HFD had a much bigger influence on the overall gut microbiota composition at the genus level than the SFP treatment. Interestingly, the distance between SFP-treated groups and the blank group in PCA plots of the predicted functional gene, metabolic pathway coverage, and abundance was much shorter than that in the plot of the gut microbiota composition (Figure 5), suggesting that SFPs may have a more pronounced influence on the function of the gut microbiota, although they only slightly altered the gut microbiota composition, especially for Sf-A and Sf-1.

### 2.5. g_Mucispinllum and g_Oscillospira Were Associated with the Fasting Blood Glucose Level

**Figure 6:**
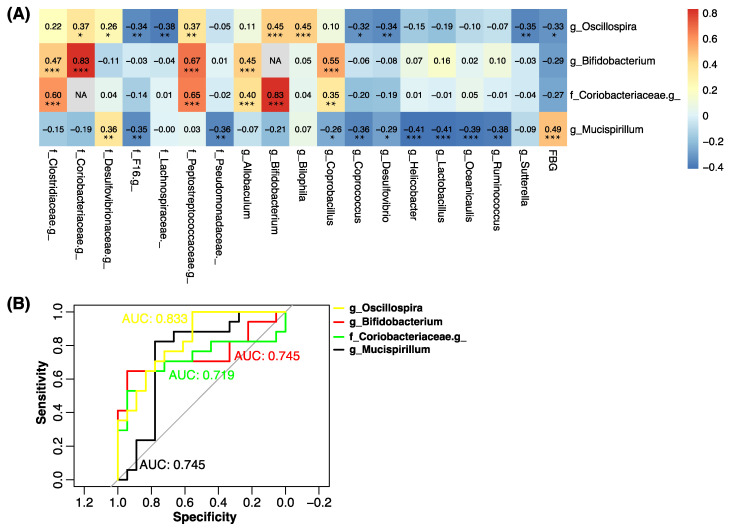
(**A**) Correlations between the fasting blood glucose level and the relative abundance of gut microbiota. A color key is shown at the bottom right of the heatmap to demonstrate the size of the correlation coefficient. Values in each lattice represent the correlation coefficients. * False discovery rate (FDR)-corrected *p*-value < 0.05; ** FDR-corrected *p*-value < 0.01; *** FDR-corrected *p*-value < 0.05. NA, not applicable. (**B**) Fitting receiver operating characteristic (ROC) curves of g_*Oscillospira*, g_*Bifidobacterium*, f_*Coriobacteriaceae*.g_, and g_*Mucispinllum* for the prediction of fasting hypoglycemia.

To gain a deeper understanding of the role of the gut microbiota in the development of hyperglycemia in HFD-treated mice, correlation analysis was carried out to explore the relationship between the FBG level and the gut microbiota abundance. The gut bacterial genera showing moderate to high correlations with the FBG and other bacterial genera showing high associations with the discovered genera were selected to prepare Figure 6. The FBG was found to exhibit a high positive correlation with the relative abundance of g_*Mucispinllum* (r = 0.49, false discovery rate (FDR)-corrected *p* = 2.2 × 10^−4^) and a moderate negative correlation with the relative abundance of g_*Oscillospira* (r = −0.33, FDR-corrected *p* = 0.028) (Figure 6). Interestingly, the relative abundance of two genera—g_*Bifidobacterium* and f_*Coriobacteriaceae*.g_ (unidentified genus belonging to f_*Coriobacteriaceae*)—not only showed a very high positive correlation with each other (r = 0.83, FDR-corrected *p* = 1.1 × 10^−18^), but also displayed high positive associations with f_*Peptostreptococcaceae*.g_ (unidentified genus belonging to f_*Peptostreptococcaceae*), g_*Allobaculum*, and g_*Coprobacillus* (r = 0.35–0.67, FDR-corrected *p* < 0.001), and moderate negative correlations with the FBG level (r = 0.27–0.29, FDR-corrected *p* < 0.09). Then, receiver operating characteristic (ROC) analysis was used to analyze the sensitivity and specificity of g_*Oscillospira*, g_*Bifidobacterium*, f_*Coriobacteriaceae*.g_, and g_*Mucispinllum* in the prediction of fasting hypoglycemia. The area under the ROC curves (AUC) of g_*Oscillospira* was 0.833, with a sensitivity and specificity of 0.556 and 1.00, respectively. The AUCs of the other three genera were also larger than 0.7 (ranging from 0.719 to 0.745). The findings indicated that g_*Oscillospira* and g_*Mucispinllum* exhibited good discriminatory power in the prediction of fasting hypoglycemia.

## 3. Discussion

Fasting hypoglycemia is a common symptom in diabetes and has also been reported in the HFD-fed animal model, but the underlying mechanism or the effect of anti-hyperglycemic products on the fasting hypoglycemia has not been investigated. SFPs can alleviate HFD-induced hyperglycemia, but the effect of SFP on HFD-induced early fasting hypoglycemia is critical for their clinical application and has not been evaluated. In the present study, five SFPs were prepared from *S*. *fusiforme* and their effects on HFD-induced fasting hypoglycemia and gut microbiota dysbiosis were investigated. The results indicated that C57BL/6J male mice fed an HFD for 4 weeks developed severe hypoglycemia and four SFPs significantly prevented early fasting hypoglycemia, without inducing hyperglycemia. SFP administration mainly attenuated the HFD-induced decrease in Bacteroidetes and increase in Actinobacteria, and had a considerable influence on the relative abundance of six genera, including *Oscillospira*, *Mucispirillum*, and *Bifidobacterium*. Correlation analysis revealed that the fasting blood glucose level is closely associated with the relative abundance of g_*Mucispinllum* and g_*Oscillospira*.

HFD-induced fasting hypoglycemia is less frequently reported than HFD-induced hyperglycemia, but hypoglycemia is a common symptom in patients with diabetes. A previous study also demonstrated that fasting hypoglycemia is associated with disease progression toward insulin-dependent type 1 diabetes and could be used as a marker for type 1 diabetes [16]. Therefore, we speculated that the HFD-induced early fasting hypoglycemia is mainly due to excessive insulin as a response to the HFD. Of note, studies have shown that a dietary fiber diet may reduce hypoglycemia by regulating the gut microbiota to reduce insulin secretion [17,18]. In this study, *S. fusiforme* polysaccharides were found to prevent HFD-induced early fasting hypoglycemia, which would be beneficial to people at a high risk of developing insulin-dependent diseases.

The effects of SFPs on the gut microbiota composition have been reported by other researchers [12,13]. Chen et al. found that SFPs could partially rejuvenate the overall status of the small intestine microbiota in mice during the aging process, including alleviating the increase in Firmicutes and decrease in Proteobacteria [12]. Cheng et al. reported that SFPs significantly altered the gut microbiota in the feces of streptozotocin-induced diabetic mice and decreased the relative abundances of diabetes-related intestinal bacteria, such as *Oscillibacter*, *Ruminococcaceae*, and *Peptostreptococcaceae* [13]. In addition, Zhang et al. found that an *Edgeworthia gardneri* (Wall.) Meisn. water extract with anti-diabetic activity can significantly alleviate the decrease in the relative abundance of o_*Clostridiales* [19]. In the present study, we found that SFPs mainly regulated the relative abundance of Actinobacteria (phylum level), and o_*Clostridiales*, *Oscillospira*, *Mucispirillum*, and *Bifidobacterium* (genus level). These findings indicate that the regulatory effects of SFPs on gut microbiota are partially consistent with previous studies, and the inconsistency may result from differences in the polysaccharide preparation, sampling location, and animal model. Moreover, the effects of SFP intervention on predicted functional gene and metabolic pathways were also discussed, which would be helpful for developing a comprehensive understanding of the roles of SFPs in gut microbiota. However, this finding needs functional validation.

Correlation analysis has been usefully applied to identify potential biomarkers from the gut microbiota in metabolic disease [20,21]. The gut microbiota can regulate blood glucose and has been considered to be a potential mechanism for the antidiabetic effect of *S. fusiforme* fucoidan [22]. In the present study, the regulatory effects of the five SFPs on the FBG level and gut microbiota composition were different. To explore the relationship between the FBG level and the gut microbiota abundance, Pearson’s correlation analysis was performed and revealed that the FBG exhibited a high positive correlation with the relative abundance of g_*Mucispinllum* and a moderate negative correlation with the relative abundance of g_*Oscillospira*, g_*Bifidobacterium*, and f_*Coriobacteriaceae*.g_. Moreover, the relative abundance of g_*Bifidobacterium*, and f_*Coriobacteriaceae*.g_ also showed a very high positive correlation with that of f_*Peptostreptococcaceae*.g_, g_*Allobaculum*, and g_*Coprobacillus*. These findings suggest that the FBG is closely associated with the relative abundance of these seven genera of gut bacteria, especially for g_*Mucispinllum* and g_*Oscillospira*. A recent study has shown the FBG levels displayed a positive correlation with the relative abundance of o_*Clostridiales*._._ and a negative correlation with the relative abundance of g_*Oscillospira* (*p* < 0.01), and fasting insulin levels showed a positive correlation with the relative abundance of g_*Oscillospira* [19], which was consistent with our findings and also confirmed the suggestion that g_*Oscillospira* may play an important role in relieving HFD-induced early hypoglycemia by SFPs. However, the detailed mechanism requires further investigation.

The five SFPs had different impacts on the FBG, which may be partially explained by their selective enrichment of the FBG-associated bacteria. The selective modulation of the gut microbiota and other activities, such as the alleviation of obesity and glycometabolic disorder, by the SFPs may be ascribed to the difference in their chemical structure. Since the five SFPs displayed multiple differences in the chemical composition or structural characteristics, it is difficult to conclude the precise structure–activity relationship of SFPs. Future in-depth structure–activity relationship studies should rely on the preparation of polysaccharides with only one difference in the chemical structure.

In conclusion, we prepared and characterized four SFPs, which could significantly prevent HFD-induced early fasting hypoglycemia and regulate the gut microbiota composition, among which, Sf-A could significantly prevent HFD-induced weight gain, maintain blood glucose homeostasis, and alleviate dysbiosis of the gut microbiome, suggesting the priority of Sf-A to be developed for health care products. Moreover, the blood glucose level was found to be associated with the relative abundance of g_*Mucispinllum* and g_*Oscillospira*, and ROC analysis indicated that g_*Oscillospira* has good discriminatory power in the prediction of fasting hypoglycemia. However, the detailed interaction of the microbes with glucose homeostasis warrants further investigation. Our findings highlight the novel application of *S. fusiforme* polysaccharides in glucose homeostasis.

## 4. Materials and Methods

### 4.1. Materials

The brown algae *S*. *fusiforme* was collected in Qingdao, China, on 28 May 2014. Standards of monosaccharides (mannose, l-rhamnose, d-glucuronic acid, d-glucose, d-galactose, d-xylose, and l-fucose) and 1-Phenyl-3-methyl-5-pyrazolone (PMP) were purchased from Aladdin Chemistry Co., Ltd. (Shanghai, China). Dextran standards were purchased from American Polymer Standards Corporation (Mentor, OH, USA). Other reagents and solvents were of analytical grade.

### 4.2. Preparation and Purification of SFPs

SFPs were prepared according to our previous study, with minor modifications [23]. Briefly, dry *S*. *fusiforme* was cut into pieces and treated with 95% ethanol three times to remove the pigment. The crude polysaccharide was extracted from the residual material using hot water or 0.1 M HCl, respectively, and then filtered with Celite and concentrated. The hot water-extracted crude polysaccharide was further treated with 20% ethanol containing 0.05 M MgCl_2_ to eliminate the alginate. After removing the alginate, the supernatant was ultra-filtered. Finally, the dialysate was concentrated and crude polysaccharide was obtained using ethanol precipitation. Then, the water-extracted crude polysaccharide was fractionated by the DEAE Sepharose Fast Flow column, and three polysaccharide fractions were obtained from elution with water (Sf-1), 0.5 M NaCl (Sf-2), and 2 M NaCl (Sf-3). Sf-3 was degraded by 0.54 M hydrogen peroxide and 0.52 M ascorbic acid, according to a previous study [24]. The degraded polysaccharide (Sf-3-1) was dialyzed and lyophilized. The HCl-extracted crude polysaccharide was dialyzed and precipitated using ethanol to obtain the Sf-A.

### 4.3. Chemical Analysis of SFPs

The total sugar content was measured by the phenol-sulfuric acid method, using d-glucose as the standard [25]. The content of uronic acid was measured according to the method described by Blumenkrantz and Asboe-Hansen [26] using d-glucuronic acid as the standard. The protein content was evaluated by the Bradford method, using BSA as the standard [27]. The content of sulfate was analyzed with the BaCl_2_-gelation method, using Na_2_SO_4_ as the standard [28]. The molecular weight analysis was conducted using High-Performance Size Exclusion Chromatography (HPSEC), employing a Waters 2487 HPLC system with a refractive index detector 2414 (Waters, Milford, MA, USA). The chromatography conditions were as follows: Eluent, 0.1 mol/L NaNO_3_; flow rate of 0.5 mL/min; two columns of TSK G5000PWXL and G3000PWXL in series. The calibration curve was plotted using the retention times of dextran standards of different molecular weights.

The monosaccharide composition analysis was carried out by the PMP derivatization method, with minor modifications [29]. Briefly, 30 mg of the crude sample was dissolved in 3 mL of 2M trifluoroacetic acid (TFA), and hydrolyzed at 110 °C for 4 h. Then, the hydrolyzed mixture was neutralized to pH 7 with sodium hydroxide. This mixture was subjected to monosaccharide PMP derivatization at 60 °C for 1 h and cooled to room temperature. Subsequently, the reaction was stopped by adding 0.3 mol/L HCl solution (200 µL), PMP was removed by extraction with chloroform, and the aqueous layer was filtered through a 0.45 μm membrane and analyzed by HPLC on an Eclipse XDB-C18 column with UV detection.

### 4.4. Animal Study

Eighty-four six-week-old male C57BL/6J mice (18–22 g) were purchased from the SPF (Beijing) Biotechnology Co.; Ltd. (Beijing, China). All mice were fed a standard lab chow diet (4% kcal fat, no cholesterol, #MD12033, Jiangsu Medicience Co., Ltd., Yangzhou, China) for one week for adaptation, and were then randomly divided into seven groups, with each group consisting of twelve animals. Group 1 (blank) still received a standard lab chow diet during the experiment and served as the blank group. The remaining groups, including the control and five SFP-treatment groups, were fed a high-fat diet (60% kcal fat, 279.6 mg/kg cholesterol, D12492, Research Diets, Inc., New Brunswick, NJ, USA) to induce experimental metabolic syndrome. Treatments were administered intragastrically once a day for 4 weeks, as follows: Group 2 was treated with distilled water and assigned as the control group, and Group 3 to Group 7 were treated with Sf-1, Sf-2, Sf-3, Sf-3-1, and Sf-A, respectively, and referred to as the Sf-1, Sf-2, Sf-3, Sf-3-1, and Sf-A group, respectively. To figure out which samples had a better efficacy in alleviating HFD-induced metabolic disorder, a relatively low dosage (100 mg/kg body weight/day) of polysaccharides was orally administered on a daily basis.

Body weight was measured weekly and fasting blood glucose (FBG) was determined via a tail vein using a glucometer (Johnson and Johnson, New Brunswick, NJ, USA), according to the instruction after fasting for 16 h. At the end of the diet period, an oral glucose tolerance test (OGTT) was also performed, according to a previous study [30]. After a 16-h fast, the animals were orally gavaged with 2 g/kg body weight of glucose solution. Blood glucose levels were measured at 0, 20, 60, and 120 min via a tail vein using a glucometer (Johnson and Johnson, USA). The cecum contents were collected and stored at −80 °C immediately after collection. The abdominal fat, liver, and pancreas were harvested and their weight was measured. All experiments were performed following the protocol for animal study approved by the Ethics Committee of Zhejiang University of Technology (20180926049).

### 4.5. DNA Extraction and High Throughput Sequencing

Gut microbial genomic DNA was extracted from the cecum contents by using a TIANamp Stool DNA Kit (DP328, Tiangen), according to the manufacturer’s instruction. The V3-V4 region of the 16S rRNA gene was amplified using the well-established universal primers 338F (5′-ACTCCTACGGGAGGCAGCA-3′) and 806R (5′-GGACTACHVGGGTWTCTAAT-3′). The amplification was performed as follows: Initial denaturation for 3 min at 95 °C, 30 cycles each of denaturation for 30 s at 95 °C, annealing for 30 s at 55 °C, and primer extension for 45 s at 72 °C. The amplicons were purified using the Ampure XP beads (A63881, Beckman Coulter, Brea, CA, USA) and quantified with a Qubit 3.0 fluorometer using a Qubit dsDNA HS Assay Kit (Q32854, Invitrogen, Carlsbad, CA, USA), before being sequenced on an Illumina Miseq PE300 platform by Hangzhou Kaitai Biotechnology Co.; Ltd. (Hangzhou, China).

### 4.6. Bioinformatics Analysis

The raw demultiplexed sequences were analyzed using QIIME2 (Quantitative Insights Into Microbial Ecology 2, v2018.4), which is a plugin-based microbiome analysis platform [31]. The sequenced reads were denoised and quality filtered with DADA2 using the q2-dada2 plugin, which removes low-quality sequences (average quality score <25 in every 50 bp sliding window), primer sequences, and chimeric sequences, and retains unique de novo sequence variants. The remaining unique sequences were taxonomically classified using classify-sklearn [32] against the Greengenes 13_8 99% OTU reference sequences [33]. Finally, a feature table was generated for relative abundance plots and further analysis. α-Diversity indices (Abundance-based Coverage Estimator (ACE) metric, and Chao1, Simpson’s and Shannon’s diversity indices) were calculated using the QIIME2 pipeline. Functional metagenomes were predicted based on the 16S rRNA sequencing data of the fecal microbiota using PICRUSt 2.0 [34].

Microbiome data were analyzed according to our previous study [35], with minor modifications. Briefly, data processing, normalization, scaling, and multivariate analyses were performed using the R package MetaboAnalystR [36]. Features with at least 50% missing values were removed and the remaining missing values were replaced with a small value. Data were further normalized to the total intensity, followed by Pareto scaling, in order to obtain normally distributed variables. Principal components analysis (PCA) was performed to assess changes of the gut microbiota, predicted functional gene family, metabolic pathway coverage, and abundance between groups. Discriminative features were selected by STAMP (STatistical Analysis of Metagenomic Profiles) [37].

### 4.7. Statistical Analysis

The significance of the differences between groups was determined using one-way ANOVA, followed by Student’s *t*-test (GraphPad Software, San Diego, CA, USA), and a *p*-value of less than 0.05 was considered significant. Pearson’s correlation analysis was carried out to evaluate the relationship between the FBG level and the gut microbiota abundance using the R package psych, and FDR-corrected *p* values of less than 0.05 and an absolute value of Pearson correlation coefficient of more than 0.3 were considered significant. An ROC curve was applied to analyze the sensitivity and specificity of selective gut bacterial genera in the prediction of fasting hypoglycemia. Only mice with blood glucose levels at the upper and lower quartiles of the range were included for ROC analysis. The area under the ROC curve was used to assess the ROC effect.

### 4.8. Accession Number

Raw sequencing data are available in the NCBI SRA BioProject database under accession no. PRJNA604399.

## Figures and Tables

**Table 1:** Physicochemical properties of *Sargassum fusiforme* polysaccharides.

Name	Total Sugar Content	Sulfate Content	Protein Content	Uronic Acid Content	Average Molecular Weight	Neutral Monosaccharide Composition (Molar Ratio)				
						Man	Gal	Glc	GlcA	Fuc
Sf-1	80.30 ± 1.03	-	1.00 ± 0.43	0.17 ± 0.25	698.3/8.9	-	-	1	-	-
Sf-2	63.73 ± 4.56	11.12 ± 0.68	1.03 ± 0.40	7.71 ± 1.56	95.5/9.5	0.26	0.23	0.21	0.30	1
Sf-3	69.23 ± 2.51	35.08 ± 1.15	2.35 ± 0.27	2.34 ± 1.09	229.5	-	0.24	-	-	1
Sf-3-1	51.95 ± 0.33	30.52 ± 1.58	0.96 ± 0.05	2.37 ± 0.66	10.0	0.09	0.33	-	0.08	1
Sf-A	63.60 ± 12.71	22.95 ± 1.77	0.47 ± 0.16	2.89 ± 0.35	46.5/5.1	0.10	0.42	1.26	0.05	1

-, not dateable.

## Supplementary Materials

The following are available online at https://www.mdpi.com/1660-3397/18/9/444/s1. Figure S1. HPSEC profile of (A) Sf-1, (B) Sf-2, (C) Sf-3, (D) Sf-3-1, (E) Sf-A. Figure S2 Effects of *S. fusiforme* polysaccharides on body weight in HFD-treated mice. Values are mean ± SEM (n = 10).
