# Supplementary material for: Sargassum fusiforme Polysaccharides Prevent High-Fat Diet-Induced Early Fasting Hypoglycemia and Regulate the Gut Microbiota Composition
Source: Mar Drugs. 2020 Aug 27;18(9):444. doi: 10.3390/md18090444 (PMC7551101; doi:10.3390/md18090444)
Supplement: Supplementary file 1 [file marinedrugs-18-00444-s001.pdf]

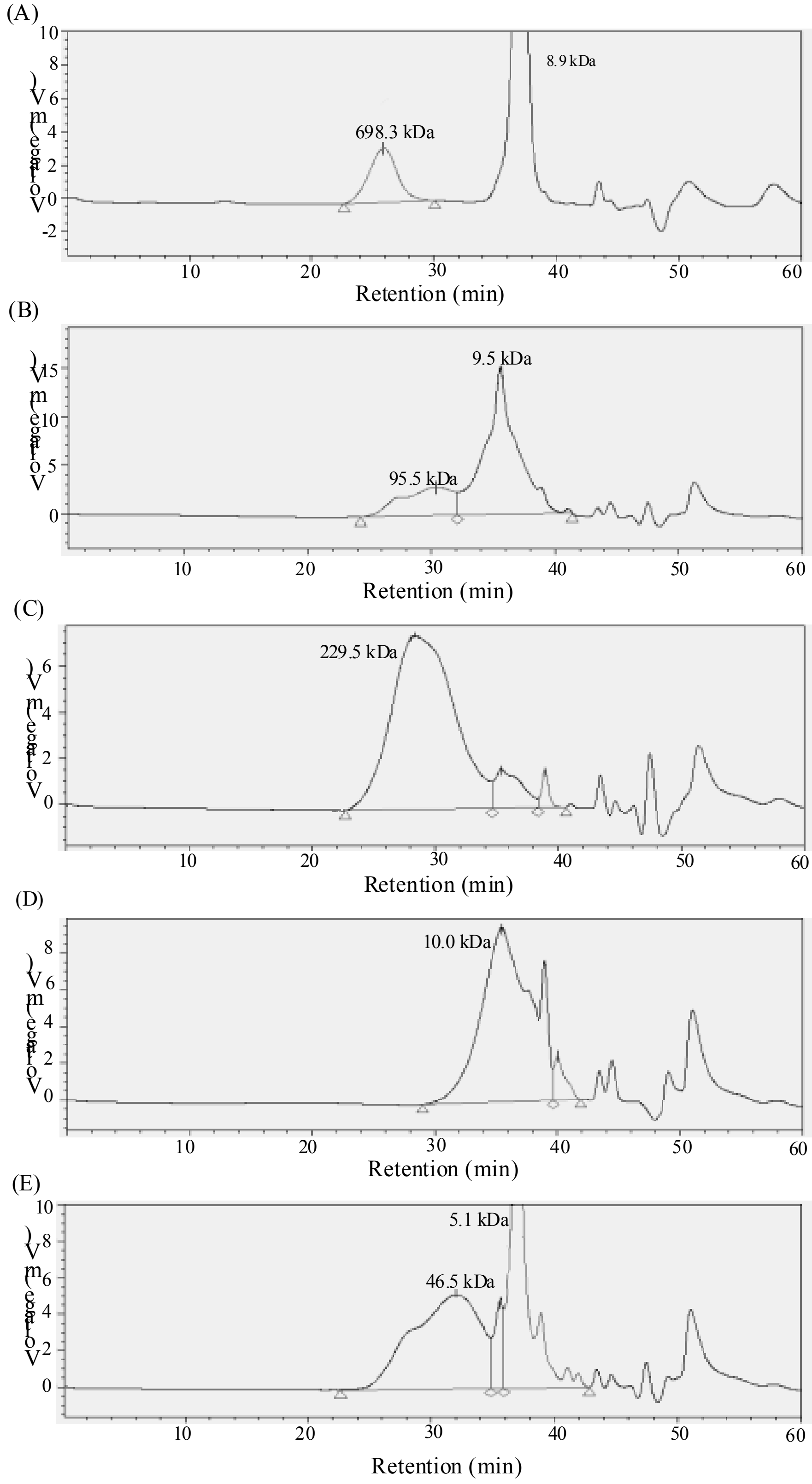

Figure S1. HPSEC profile of (A) Sf-1, (B) Sf-2, (C) Sf-3, (D) Sf-3-1, (E) Sf-A.

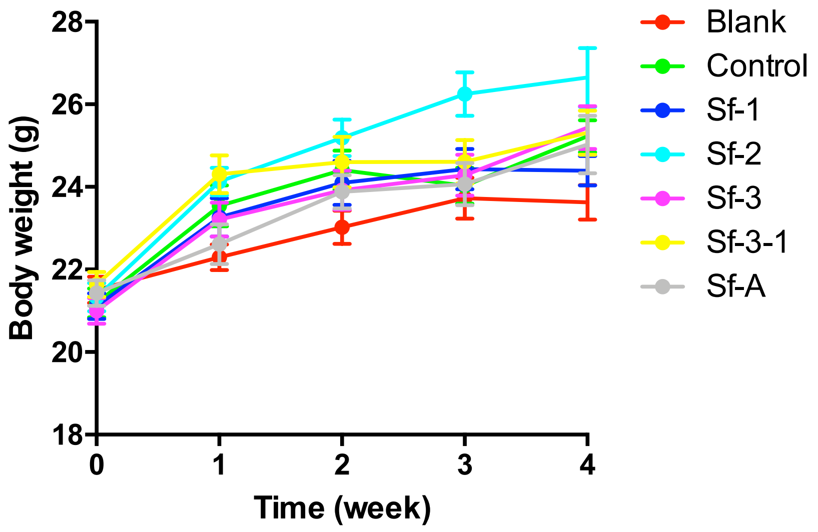

Figure S2 Effects of *S. fusiforme* polysaccharides on body weight in HFD-treated mice. Values are mean ± SEM (n=10).
